# Photobleaching and Fluorescence Recovery of RPE Bisretinoids

**DOI:** 10.1371/journal.pone.0138081

**Published:** 2015-09-14

**Authors:** Zhao Liu, Keiko Ueda, Hye Jin Kim, Janet R. Sparrow

**Affiliations:** 1 Department of Ophthalmology, Columbia University Medical Center, New York, New York, United States of America; 2 Departments of Pathology and Cell Biology, Columbia University Medical Center, New York, New York, United States of America; Eye Hospital, Charité, GERMANY

## Abstract

The autofluorescence of the retina that originates primarily from lipofuscin fluorophores in retinal pigment epithelial cells, is observed to undergo photobleaching during the acquisition of fundus autofluorescence images. Bisretinoid fluorophores isolated from retinal pigment epithelial cells have the spectral characteristics consistent with their being the source of fundus autofluorescence. Clinically relevant experiments were designed to better understand conditions in the micromilieu of bisretinoid fluorophores that can influence fluorescence efficiencies, photobleaching, and subsequent fluorescence recovery of this fluorophore. The consumption of the bisretinoid A2E due to photooxidation-induced degradation was quantified in solvent systems of variable relative permittivity (formerly called dielectric constant), in micelles, and in phospholipid vesicles of varying composition. Reorganization within biphasic systems was also examined. A2E content was measured by high performance liquid chromatography (HPLC) and fluorescence intensity was quantified spectroscopically. As solvent polarity was increased, A2E fluorescent spectra exhibited red-shifted maxima and reduced intensity. A2E was depleted by light irradiation and the loss was more pronounced in less polar solvents, lower concentrations of anionic surfactant, and in gel- versus fluid-ordered phospholipid liposomes. Conditions that permit A2E aggregation promoted photooxidation/photodegradation, while movement of A2E between bisphasic systems was associated with fluorescence recovery after photobleaching. The fluorescence characteristics of A2E are subject to environmental modulation. Photooxidation and photodegradation of bisretinoid can account for fundus autofluorescence photobleaching. Return of fluorescence intensity after photobleaching likely occurs due to redistribution of A2E fractions amongst co-existing heterogeneous microdomains of the lysosomal compartment.

## Introduction

Fluorescence imaging of the inherent autofluorescence of retinal pigment epithelial (RPE) lipofuscin by adaptive optics scanning laser ophthalmoscopy (AOSLO) [1, 2] has permitted individual cells of the RPE monolayer to be viewed. Early studies in non-human primates revealed interesting fluorescence and cellular changes [1–3]. In particular, when light was delivered at 40 μW (210 J/cm^2^) power and an excitation of 568 nm, the natural autofluorescence of the RPE monolayer was noticeably diminished (~20% decrease) immediately after the exposure. Subsequently, autofluorescence fully recovered within hours and there was no sign of cellular damage. Similar photofading (bleaching) was observed with 488 nm excitation [3]. On the other hand, at average powers of 55 and 150 μW (289 and 788 J/cm^2^, respectively), levels that were still considered safe by light safety standards, autofluorescence was reduced in the exposed area by as much as 42%. After 6 days, cellular disruption was observed in the same location. Imaging with the SLO without AO produced the same autofluorescence bleaching and RPE damage. Photobleaching similar to the RPE autofluorescence reduction *in vivo* was observed by employing cell-associated A2E as a bisretinoid model of RPE lipofuscin behavior [4]. With this cell model, exposures conferring a bleach of approximately 16% permitted subsequent recovery of autofluorescence. Fluorescence bleaching of bisretinoid was shown to involve photooxidative and photodegradative processes and the potential for autofluorescence recovery was observed to depend on light dose and antioxidant status.

Photobleaching is the loss of fluorescence that is typically caused by interactions amongst a photosensitizer molecule, light of an appropriate wavelength, and oxygen [5]. When a photosensitizer absorbs a photon, the excited electron is promoted to the first excited singlet state (electron spins of the ground state and excited electrons are paired and opposite) from which the electron can dissipate the excess energy by processes such heat and fluorescence emission or by intersystem crossing to the triplet excited state (the spin of the excited electron is reversed). The latter process is possible for molecules like A2E that have conjugated double bond systems; these π–electron systems also permit long-lived triplet states. The excited sensitizer in the triplet state may react either by hydrogen-atom abstraction or electron-transfer with a substrate to form active oxygen species such as superoxide radical anion or it can transfer energy to molecular oxygen producing excited state singlet oxygen (^1^O_2_). Preference for any of these forms of deactivation depends on the properties of a particular molecule.

As with some other photosensitive compounds [6], A2E can also serve as the substrate for the reaction of singlet oxygen and radical oxygen species at double bonds; through this process A2E itself is photobleached [7, 8]. Evidence for the participation of singlet oxygen in bisretinoid photooxidation is available from several lines of investigation. For instance, in experiments with A2E in which singlet oxygen lifetime was extended by replacing the H_2_O solvent with D_2_O [9], A2E photodegradation efficiency was increased [8]. On the other hand, photodegradation was reduced in deoxygenated solutions and by the addition of the singlet oxygen quencher 1,2,2,6,6-pentamethyl-4-piperidinol (NMP). A2E oxidation was also observed when an exogenous singlet oxygen generator substituted for short-wavelength irradiation [9]. Additionally, photodestruction of A2E is known to occur at sites of endoperoxide moieties [10] that form secondary to cycloaddiiton of singlet oxygen [11]. Indeed, experiments aimed at measuring the quantum yield of singlet oxygen production by A2E [12, 13], have been confounded by the propensity for A2E to also serve as a substrate for reaction with singlet oxygen.

**Fig 1:**
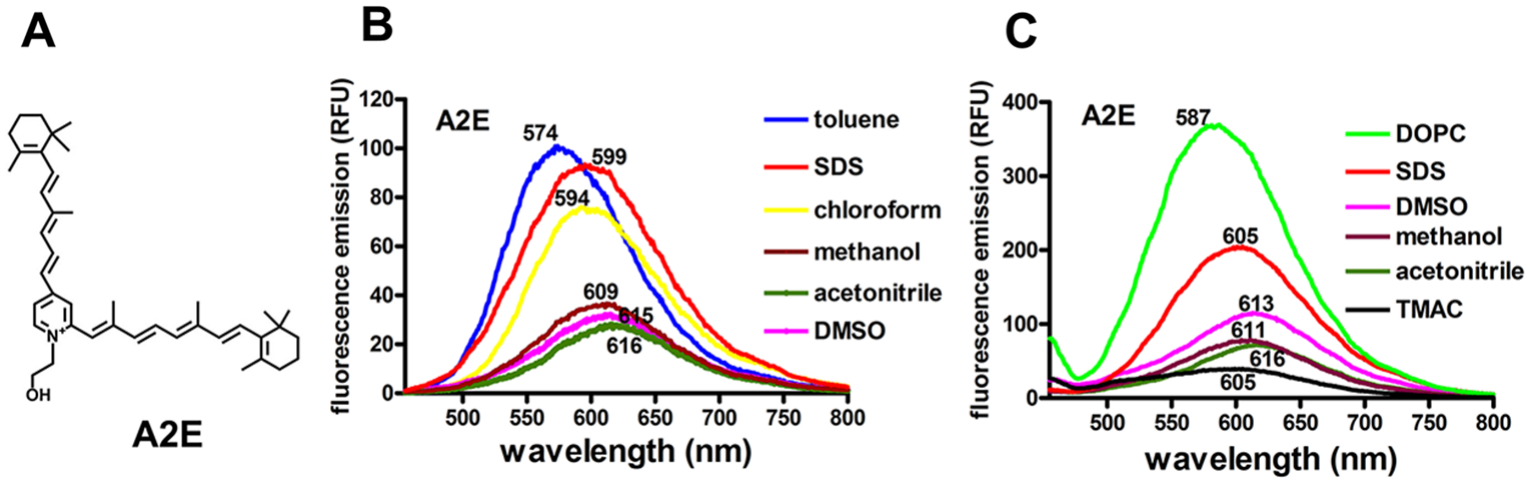
Fluorescence emission spectra of A2E. (A) Structure of the RPE lipofuscin fluorophore A2E. (B) Emission spectra of A2E in various solvents (toluene, chloroform, methanol, acetonitrile and 100% dimethylsulfoxide, DMSO) and in the surfactant sodium dodecyl sulfate (SDS, 1mM) were recorded from a quartz cuvette. Fluorescence (relative fluorescence units, RFU) was recorded at an excitation of 440 nm and A2E concentration of 100 μM. Emission maximum (nm) of each spectral profile is indicated. (C) Fluorescence emission spectra of A2E in 1,2-dipalmitoyl-*sn*-glycero-3-phosphatidylcholine (DOPC 1 mM), SDS, DMSO, methanol, acetonitrile and trialkyl-methylammonium chloride (TMAC, 1 mM) recorded from a 384-well microplate.

The fluorophores of RPE lipofuscin are sequestered within lysosomal organelles of the cell. To explain the autofluorescence changes observed in primates and in cellular systems [1, 4], we propose a model within which bisretinoids of RPE lipofuscin self-organize into microdomains that influence their photoreactivity and fluorescence efficiency. Movement between these systems could potentially play a role in photobleaching recovery. To test this model we have utilized A2E, one member of a multipart mixture of RPE lipofuscin fluorophores. A2E is a quaternary amine with the nitrogen covalently bound to four carbon atoms [14, 15] (Fig 1A). The positive charge on the nitrogen atom is not pH-dependent but is permanent, and like other quaternary amines, the cation binds an anion (probably chloride) to form a salt. From this pyridinium head group two polyene hydrophobic arms are extended, each of which serves as a chromophore: the 439 nm absorbance in the visible spectrum is generated by the long arm and a 335 nm absorbance originates from the short arm [16]. As is typical for photosensitizers, the arms of A2E consist of systems of conjugated double bonds containing delocalized electrons within orbitals that extend over several adjacent atoms; these systems allow for both electron and energy transfer and they confer lower oxidation potential. The central objective of the current study was to better understand molecular mechanisms underlying the loss of and recovery of RPE autofluorescence so as to evaluate these processes relative to risk of RPE cell photodamage.

## Materials and Methods

### A2E synthesis

A2E was synthesized as previously described [17] and was purified using an Alliance HPLC system (Waters Corp., Milford, MA) with a reverse phase column (C18, 40×250 mm, 5 μm). The eluting acetonitrile/water (containing 0.1% trifluoroacetic acid) gradient was: 84–100% (0–20 min), 100% acetonitrile (20–40 min), with a flow rate of 2.3 mL/min and monitoring at 430 nm.

### Irradiation

Irradiation was performed by delivering light of 430 nm (± 20 nm) from a tungsten halogen source (0.18 mW/cm^2^) for the times indicated. Irradiance levels were measured with a Newport Optical Power meter (Model 840, Newport Corp., Irvine CA).

### Spectroscopy

Fluorescence spectra of A2E in indicated solvents and liposomes (1 mM DOPC) were recorded in a SpectraMax M5 reader (Molecular Devices Inc, Sunnyvale CA) using either a fluorescence quartz cuvette (10 mm path-length) (Starna Cells, Inc., Atascadero, CA) or a 384-well microplate (polystyrene, flat-bottom, well depth of 11.56 mm) as indicated. Fluorescence was recorded at room temperature as relative fluorescence units from 400 to 750 nm in 1 or 10 nm-steps, a bandpass slit of 6 nm and an excitation of 440 nm.

### Solvent systems

A2E (50 μM) in organic solvent (300 μl) was irradiated with 430 nm light for the times indicated and A2E remaining after irradiation was quantified by high performance liquid chromatography (HPLC). The non-polar solvents included rapeseed oil (KIC Chemicals, Inc New Paltz NY) (relative permittivity, ~2), toluene (relative permittivity, 2.38) and chloroform (relative permittivity, 4.81). The polar solvents were methanol (relative permittivity 33) and acetonitrile (relative permittivity, 37.5), and dimethyl sulfoxide (DMSO) (relative permittivity, 46.7) were also tested. Solvents were obtained from Fisher Scientific (Pittsburg, PA); toluene, chloroform, methanol and acetonitrile were HPLC grade.

### Micellar systems

Stock A2E (5mM in DMSO) was added to phosphate-buffered saline (PBS, pH 7.4) with sodium dodecyl sulfate (SDS) or trialkyl-methylammonium chloride (TMAC) (Sigma-Aldrich, St. Louis, MO) for a final A2E concentration of 50 μM. The mixture was sonicated for 5 min to generate a clear micellar solution.

### Liposomes

Lipid vesicles were prepared from DOPC (1,2-dioleoyl-*sn*-glycero-3-phosphocholine), POPC (palmityl-oleoyl-*sn*-glycero-3-phosphocholine), DPPC (1,2-dipalmityl-*sn*-glycero-3-phosphocholine) (Avanti Polar Lipids Inc., Alabaster, Alabama). Vesicles were also prepared with a phospholipid mixture (PL mixture) consisting of DOPC, (1,2-dioleoyl-*sn*-glycero-3-phosphocholine), DOPS (1,2-dioleoyl-*sn*-glycero-3-phosphoserine) and DOPE (1,2-dioleoyl-*sn*-glycero-3-phosphoethanolamine) at the ratio of 65:25:10. Stock A2E in methanol was mixed with lipid in chloroform and dried under argon. The residual solvent was removed using a high vacuum pump for 1 hour. PBS was added to the lipid film and hydrated for 1 hour. The mixture was vortexed for 30 sec and sonicated for 30 min to generate liposomes. The final concentration of A2E was 50 μM and the total lipid concentration was 1 mM.

### Lipid vesicle/aqueous and oil phases

To prepare the upper phase of a two-phase system, stock A2E (5 mM in DMSO) was added to 30 μl oil (oil-A2E) to achieve 0.5 mM final concentration; the mixture was sonicated. For the lower phase, A2E was added to 300 μl of liposomes composed of DOPC, POPC, DPPC, and a PL mixture (final concentration 1 mM), or SDS (20 μM) in PBS. The final concentration of A2E in the vesicles was 50 μM. Samples of A2E in PBS with 1% DMSO were also used.

The lower phase was placed in a 2 ml glass vial with a Teflon cap, and was overlaid with the A2E-oil preparation without disturbing the interface. The mixture was irradiated from the bottom by 430 nm light for 30 seconds. The sample was sealed and incubated at room temperature for 16 hours. The A2E-liposomal or A2E-micelle fraction of the lower phase was analyzed by HPLC after irradiation and incubation and was quantified as peak areas at 430 nm absorbance.

For experiments in which fluorescence was recorded, the lower phase (50 μM, 1 ml) was prepared in a fluorescence quartz cuvette (1.4 ml, 10 mm length) (Starna Cells, Inc., Atascadero, CA), overlaid with the A2E-oil mixture (500 μM, 100 μl) and covered with a Teflon cap. The mixture was irradiated with 430 nm light for 20 minutes and incubated at room temperature for 16 hours. Fluorescence intensity (excitation/emission 430/600 nm) of A2E in the lower phase was read with a SpectraMax M5 reader (Molecular Devices Inc, Sunnyvale CA) before and after irradiation and incubation. In control samples the biphasic systems were prepared without irradiation.

### HPLC

A2E quantification was performed on an Alliance system (Waters Corp, Milford, MA) equipped with 2695 Separation Module, and 2996 Photodiode Array Detector and operating with Empower software. The mixture was separated by a reverse phase Atlantis® dC18 column (2.1×150 mm, 3 μm) using the following gradient of acetonitrile in water (containing 0.1% trifluoroacetic acid): 85 to 100% from 0 to 15 min and 100% acetonitrile from 15 to 25 min, with a flow rate of 0.8 mL/min and monitoring at 430 nm. The injection volume was 10 μl.

### Statistical Analysis

Statistical analysis was performed using PRISM 6 (GraphPad Software, Inc. La Jolla CA).

## Results

### A2E emission spectra

The wavelength of maximum emission and emission intensity of the bisretinoid A2E varies with the solvent system. Of the solvents we studied, A2E fluorescence intensity was most pronounced in toluene; at an excitation of 440 nm, the emission maximum in this solvent was 574 nm (Fig 1B). In the slightly less hydrophobic solvent chloroform, the emission maximum was shifted to 594 nm and was of reduced intensity. In the more polar solvents methanol and acetonitrile the emission maximum was red-shifted and of considerably less intensity. In SDS (1 mM in PBS), a negatively charged surfactant with which A2E can form micelles because of its cationic head group, the fluorescence intensity of A2E was close to that of toluene but the wavelength of maximum emission was longer (599 nm). Emission in 100% DMSO was similar to that in methanol and acetonitrile. We also recorded the fluorescence emission of A2E in DOPC (Fig 1C). As expected of a hydrophobic environment, the emission was maximal at 587 nm, a blue-shift when considered relative to SDS, but the fluorescence was considerably more intense. When introduced to TMAC (trialkyl-methylammonium chloride) a positively charged detergent with which A2E would not associate, fluorescence emission was minimal. Taken together, these spectra indicate that A2E emission maximum is shifted to shorter wavelengths when recorded from hydrophobic environments and the emission intensity is greater than in more polar solvents.

### Effect of solvent milieu on photo-bleaching of A2E

The local environment of a photoreactive compound can modulate its behavior. Thus we tested the photobleaching of A2E as a function of irradiation time in polar and non-polar solvents of varying relative permittivity. By HPLC analysis, photobleaching presents as diminished A2E content in a sample that has been exposed to light at a wavelength within the absorbance spectrum of A2E.

**Fig 2:**
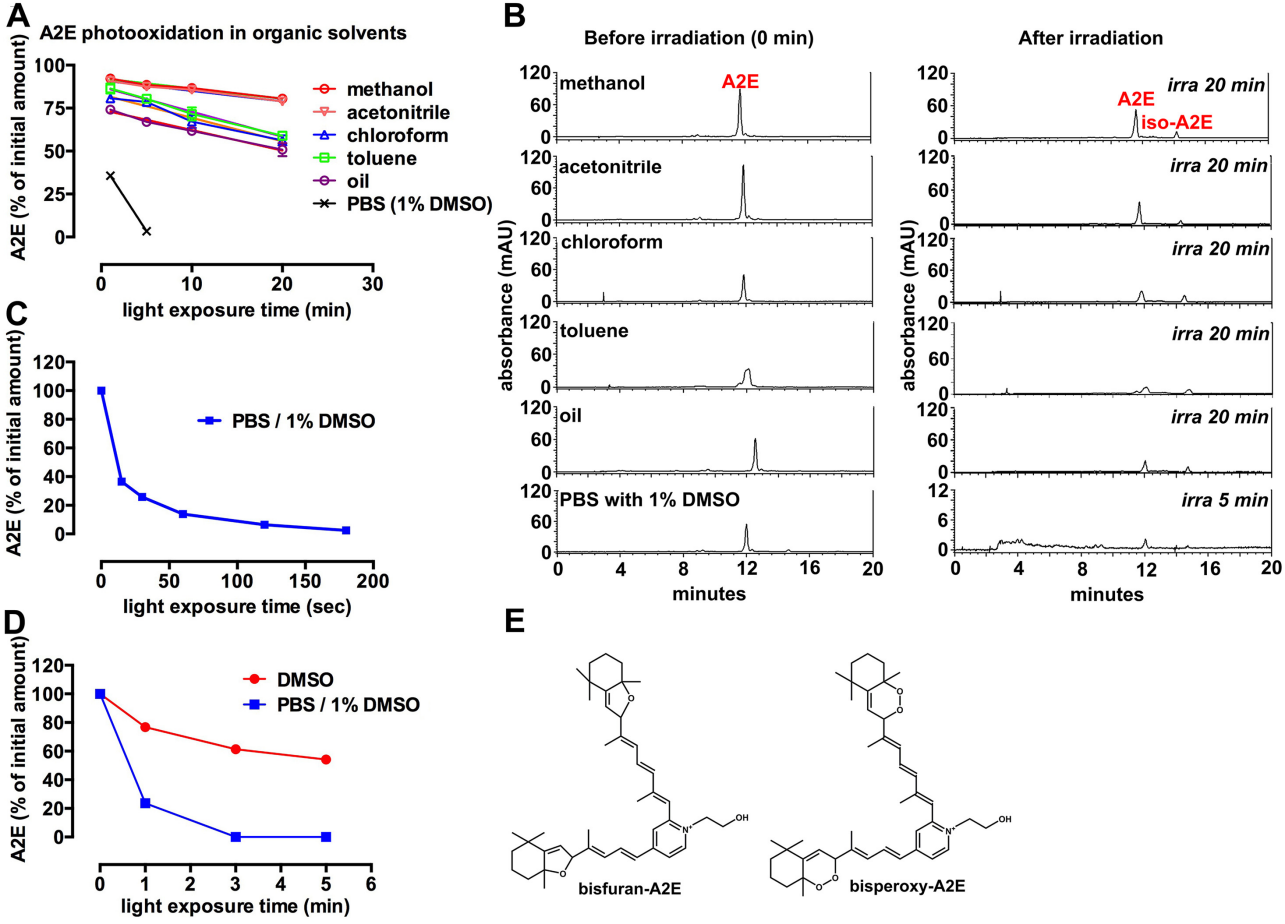
The rate of A2E photooxidation/photodegradation varies with the solvent milieu. (A) Light associated (430 nm) consumption of A2E was examined in solvents varying in polarity; in oil; and in PBS with 1% DMSO. Relative permittivities are methanol: 33; acetonitrile (37.5), chloroform: 4.8; toluene, 2.38. A2E (50 μM) in methanol, acetonitrile, chloroform and oil was irradiated for 1, 5, 10 and 20 mins and remaining A2E was analyzed by HPLC with monitoring at 430 nm. (B) Reverse phase chromatographic detection of A2E before and after irradiation in various solvents. (C) A2E (50 μM) photooxidation/photodegradation in PBS with 1% DMSO versus 100% DMSO (relative permittivity of DMSO, 49). Means (± SEM) of 3 replicates. Error bars that are not visible do not extend outside the symbol. (D) A2E (50 μM) photooxidation/photodegradation in PBS with 1% DMSO and irradiation times of 15–180 seconds. Means ± SEM of 2 replicates. (E) The structures of 2 forms of oxidized A2E.

When the amount of A2E was plotted as a percent of the starting value (Fig 2A), A2E was revealed to be depleted by light exposure and the linear relationship between residual A2E levels and time of irradiation indicated that A2E amounts were decreasing at a steady rate during exposure, though this rate varied with the polarity of the solvent. For instance, in the highly polar solvents methanol (relative permittivity, 33) and acetonitrile (relative permittivity, 37.5) there was a modest loss of A2E, and the rate of loss of A2E in these two relatively polar solvents was similar. The negative slopes (± SEM) reflected the decline in A2E as duration of irradiation was increased (acetonitrile: slope, -0.61 ± 0.05; goodness of fit, R^2^, 0.93) (methanol; -0.59 ± 0.10; goodness of fit, R^2^, 0.77). In the less polar solvents chloroform (relative permittivity, 4.80; slope, -1.36 ± 0.18; goodness of fit, R^2^, 0.85) and toluene (relative permittivity, 2.38; slope, -1.449 ± 0.1775; goodness of fit, R^2^, 0.87) the photooxidation-induced consumption of A2E occurred at a faster rate. In an oil composed of a mixture of fatty acids, the slope further increased (slope, -1.19 ± 0.15; goodness of fit, R^2^, 0.86). The slopes of the lines describing A2E bleaching in methanol and acetonitrile were not different (p > 0.05) but acetonitrile and methanol were both different than with chloroform, toluene, oil and DMSO/PBS (one-way ANOVA and Tukey’s multiple comparison test). Amongst the nonpolar solvents, the slope of the loss of A2E in chloroform was not different than that for toluene but the slopes in chloroform and toluene were different than in oil and DMSO/PBS (p > 0.05; one-way ANOVA and Tukey’s multiple comparison test). Representative chromatographs are presented in Fig 2B.

DMSO (relative permittivity, 49) is a small amphiphilic molecule that is highly miscible in both polar and non-polar milieu. In the latter case, DMSO also forms a channel of methyl groups that provide a favorable environment within which hydrophobic moieties can reside [18]. As shown in Fig 2C, in a biphasic system consisting of PBS with 1% DMSO to achieve solvation, the consumption of A2E was rapid. This observation was consistent with our previous work [9]. When A2E in PBS with 1% DMSO was irradiated and monitored at shorter exposure intervals, it was apparent that the consumption of the fluorophore followed an exponential curve (Fig 2D). In 1% DMSO/PBS, the loss of A2E was also more rapid than in 100% DMSO, perhaps because of an oxygen solubility effect (Fig 2E).

### Photo-bleaching of A2E in SDS micelle systems

**Fig 3:**
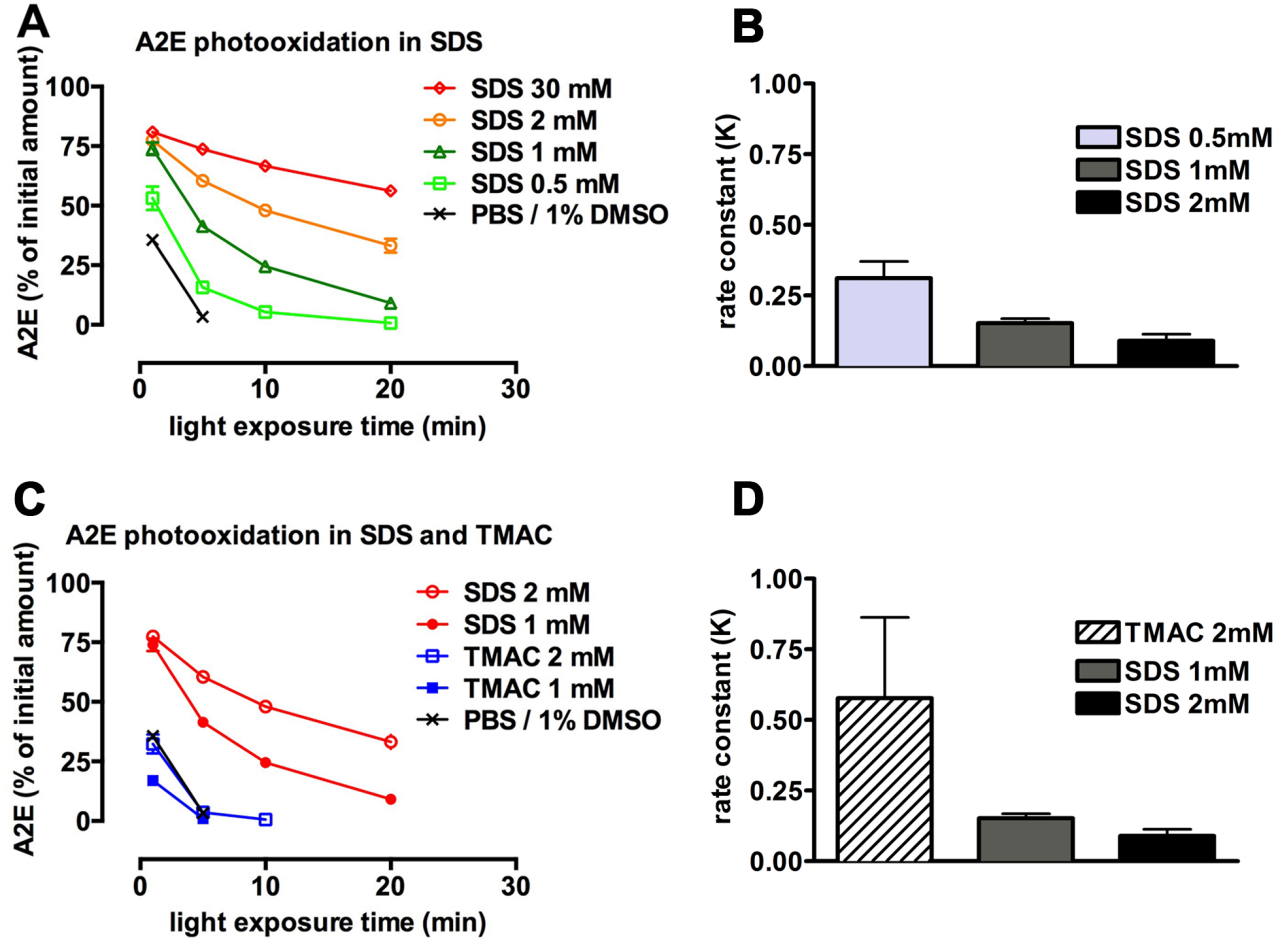
A2E photooxidation/photodegradation in micellar systems. (A) A2E (50 μM) in phosphate buffered saline containing sodium dodecyl sulfate (SDS) at concentrations indicated was irradiated for 1, 5, 10 and 20 mins and remaining A2E was analyzed by HPLC with monitoring at 430 nm. (B) Rate constants for A2E photodegradation with SDS at concentrations 0.5, 1 and 2 mM. (C) A2E photooxidation/photodegradation was compared in micellar systems composed of SDS and trialkyl-methylammonium chloride (TMAC) in PBS. A2E consumption due to photooxidation/photodegradation is also presented in PBS with 1% DMSO with irradiation for 1 and 5 minutes. (D) Rate constants with TMAC at 1 and 2 mM. Means ± (SEM) of 3 replicates. Error bars that are not visible do not extend outside the symbol.

We next investigated A2E photooxidation in an SDS/PBS system. SDS is a simple anionic surfactant consisting of a 12-carbon tail attached to a sulfate group. As a sodium salt, SDS dissociates into an anion (CH_3_(CH_2_)_11_OSO_3_) and the sodium (Na^+^) counterion when placed in an aqueous solution. A2E was expected to have an affinity for the SDS micelles because this system minimizes contact between water-based PBS buffer and the hydrophobic arms of A2E. We anticipated that A2E would readily partition into a self-assembling micellar system with SDS since its cationic head group would neutralize the repulsion between adjacent negatively charged head groups of SDS. Photodegradation at a range of SDS concentrations was monitored by HPLC measurement of the loss of A2E absorbance. We observed an inverse relationship between SDS concentration and the A2E consumption that accompanied photobleaching (Fig 3A). Specifically, as SDS concentration was increased from 0.5 mM to 2 mM, the consumption of A2E due to photooxidation and photodegradation was reduced. At SDS concentrations between 0.5 and 2 mM, the best fit of the data was a negative exponential model wherein the loss of A2E due to photooxidation at each time point was proportional to the amount remaining (Fig 3A). The half-lives were 2.2, 4.6, 7.7 and 15.4 minutes at SDS concentrations of 0.5, 1, 2 and 30 mM SDS, respectively. The rate constants for the SDS concentrations (0.5, 1, and 2 mM) associated with an exponential loss of A2E, are presented in Fig 3B.

The inverse relationship between SDS concentration and rate of photooxidation/photodegradation of A2E could reflect a local concentration effect whereby at higher SDS concentrations, A2E molecules would distribute with greater separation between the molecules. Interestingly, in the presence of TMAC (trialkyl-methylammonium chloride), a cationic surfactant (dodecyl trimethylammonium chloride), light-associated A2E consumption was rapid (Fig 3C and 3D). It is likely that TMAC, which consists of four methyl groups attached to a positively charged central nitrogen atom [19], would repel the positive charge of A2E thereby not permitting A2E to associate with the surfactant.

### Effect of liposome milieu on photo-bleaching of A2E

**Fig 4:**
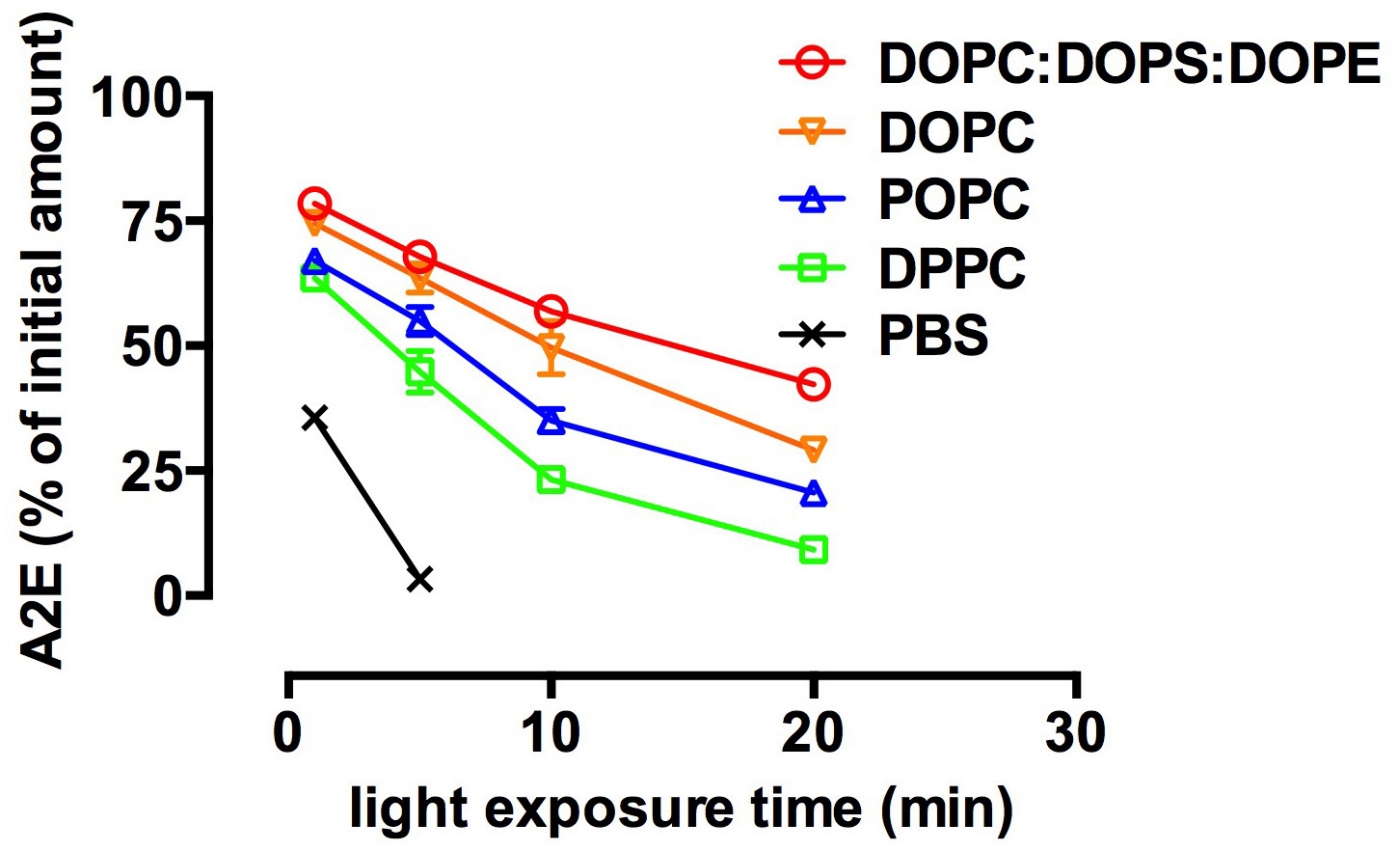
The rate of photooxidation/photodegradation of A2E varies with the composition of phospholipid liposomes. (A) A2E (50 μM) was incorporated into phosphatidylcholine vesicles having the following fatty acid chains: DPPC (1,2-dipalmitoyl-*sn*-glycero-3-phosphatidylcholine); DOPC (1, 2- dioleoyl- *sn*- glycero- 3- phosphatidylcholine); PO-PC (1- palmitoyl- 2- oleoyl- *sn*- glycero- 3- phosphatidylcholine) and to mixed vesicles of DOPC, DOPS (1, 2- dioleoyl- *sn*- glycero- 3- phosphatidylcholine); and DOPE (1, 2- dioleoyl- *sn*- glycero- 3- phosphatidylethanolamine) (ratio of 65:25:10). Vesicles were prepared in phosphate buffered saline and irradiated for 1, 5, 10 and 20 mins and remaining A2E was analyzed by HPLC with monitoring at 430 nm absorbance. Comparison was made to A2E in PBS with 1% DMSO. (B) Rate constants for A2E photodegradation in the presence of the indicated phospholipid liposomes. Means ± (SEM) of 3 replicates. Error bars that are not visible do not extend outside the symbol.

To further examine the effect of environment on A2E oxidation and degradation, we employed artificial bilayers consisting of phospholipid species (Fig 4). Fatty acid variants of the common neutral lipid phosphatidylcholine (PC) included DPPC (1,2-dipalmitoyl-*sn*-glycero-3-phosphatidylcholine) with saturated acyl chains that confer a gel-phase; DOPC (1, 2- dioleoyl- *sn*- glycero- 3- phosphatidylcholine), with unsaturated chains that bestow a fluid-phase; and POPC (1- palmitoyl- 2- oleoyl- *sn*- glycero- 3- phosphatidylcholine) that presents as coexisting fluid and gel phases. A PL mixture consisting of DOPC, DOPS and DOPE in a ratio of 65:25:10 was also employed. After irradiation, residual A2E was measured by HPLC. A2E was expressed as a percent of initial levels, was plotted as a function of irradiation time, and was analyzed by non-linear regression as shown in Fig 4A.

For A2E photodegradative loss in DPPC, POPC, and the PL mixture (DOPC: DOPS: DOPE), the F-test for model selection disclosed that a negative exponential function was the best fit (*F* ratio (degrees of freedom numerator, DFn, degrees of freedom denominator, DFd): DPPC, F(1,9) = 22.6 (*p* = 0.001); POPC, F(1,9) = 8.3 (*p* = 0.018); PL mixture, F(1,9) = 34.1, (*p* = 0.0002)), while in DOPC, the null hypothesis that the data were best described as a linear function was not rejected (F(1,9) = 0.7741, *p* = 0.41). The coefficient of determination (R^2^), values calculated as an estimate of goodness of fit of the exponential functions, were 0.97 (DPPC), 0.96 (POPC), 0.99 (PL). R^2^ under conditions of DOPC was 0.92. In each case the phospholipid environment predicted an appreciable proportion of the variance. Essentially, the photobleaching of A2E was faster in a gel-ordered lipid environment such as in DPPC vesicles and was less pronounced in a fluid-phase (e.g. DOPC vesicles). The rate constants for the phospholipid environments associated with an exponential loss of A2E under the current conditions are shown in Fig 4B.

### Movement of A2E between aqueous and oil phases

Transfer of A2E between separate domains was investigated by preparing 2-phase systems. Analysis was carried out by both HPLC and fluorescence spectroscopy. Preliminary experiments were performed to determine the photooxidation conditions that would allow the detection of a decrease in A2E and A2E fluorescence without extinguishing the signals. Differing instrumentation necessitated the use of vials of different height and volumes. As described below, we found that the fluorescence intensity of A2E was more resistant to decline than A2E abundance measured by HPLC. The latter observation could be explained by the formation of photoproducts having emission bands which overlapped with those of the parent molecule.

**Fig 5:**
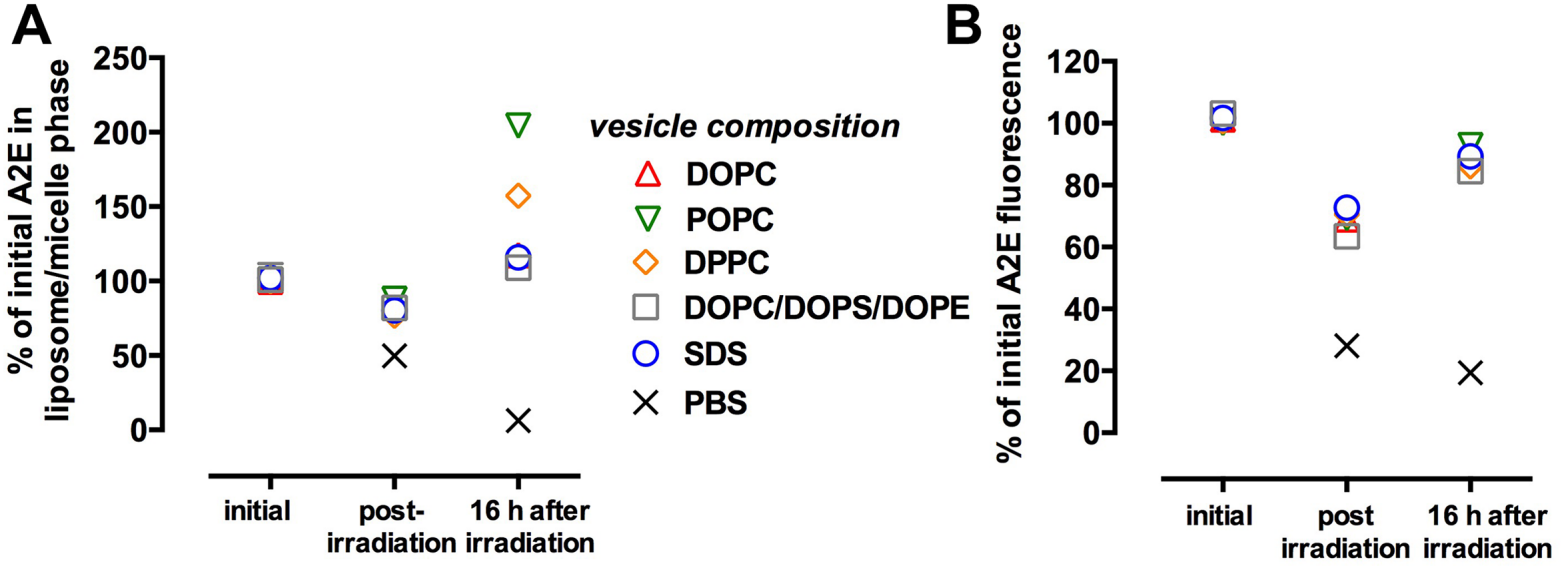
Recovery of A2E autofluorescence after bleaching depends on movement of A2E between phases. (A) HPLC measurement of A2E by HPLC. A2E in vesicles (50 μM, 300 μl) and A2E in oil (500 μM, 30 μl) occupied lower and upper phases, respectively, in a 2 ml glass vial. The lower phase was irradiated (430 nm), the biphase-systems were incubated for 16 hours as indicated and then lower phase analyzed by HPLC. (B) Measurement of A2E fluorescence by spectrofluorometry. A2E in vesicles (50 μM, 1 ml) and A2E in oil (500 μM, 100 μl) occupied lower and upper phases, respectively of a quartz cuvette (1.4 ml). Lower phase was irradiated (430 nm, the biphase-systems were incubated for 16 hours as indicated then then the lower phase was analyzed by spectrofluorometry. DPPC (1,2-dipalmitoyl-*sn*-glycero-3-phosphatidylcholine); DOPC (1, 2- dioleoyl- *sn*- glycero- 3- phosphatidylcholine); POPC (1- palmitoyl- 2- oleoyl- *sn*- glycero- 3- phosphatidylcholine) and to mixed phospholipid vesicles of DOPC, DOPS (1, 2- dioleoyl- *sn*- glycero- 3- phosphatidylcholine); and DOPE (1, 2- dioleoyl- *sn*- glycero- 3- phosphatidylethanolamine) (ratio of 65:25:10). Means ± (SEM) of 3 replicates. Error bars that are not visible do not extend outside the symbol. Symbols indicating initial measurements overlapped.

In the first set of experiments, A2E in the lower phase was distributed amongst liposomes composed of DOPC, POPC, DPPC, and a PL mixture in PBS. After irradiating (430 nm) the lower phase and further incubation, A2E in this phase was analyzed by HPLC and plotted as a percent of the starting levels (time 0). As shown in Fig 5A, when the lower phase consisted of A2E introduced to liposomes composed of DOPC, POPC, DPPC, or the PL mixture in PBS, A2E levels in the layer were always decreased by 20–30% immediately after 430 nm irradiation. However after incubation at room temperature for 16 hours, A2E levels were replenished to 108%-203% of the starting amounts. The most pronounced recovery of A2E amounts occurred when the lower layer consisted of POPC (203% ± 0.33%), the lipid that confers a gel/liquid ordered phase. Even in the absence of irradiation, A2E levels in the lower phase were increased after overnight incubation (data not shown). Interestingly however, when the lower phase consisted of A2E in DMSO/PBS, the levels of A2E in the lower phase not only declined after irradiation, but also did not revert and instead continued to decrease during the 16 hour incubation after irradiation.

We also studied movement of A2E between phases when the two systems were composed of SDS-A2E (lower phase) and oil-A2E (upper phase) (Fig 5A). Again, A2E in the lower phase was decreased immediately after this domain was irradiated; levels of A2E rose to 114% (± 2.6%) of starting levels after overnight incubation. The increase in SDS-associated A2E in the lower layer also increased after incubation in the absence of previous irradiation.

Irradiation associated-loss and recovery of A2E fluorescence was also observed within these two-phase systems. It is apparent from Fig 5B that in the absence of irradiation, the fluorescence intensity of A2E, measured as arbitrary units of the detector, varied with the particular milieu. Nevertheless, in all phospholipid vesicle preparations, photobleaching was evidenced by a post-irradiation decrease in fluorescence intensity of 30–40% relative to initial fluorescence intensity. The greatest percent decrease was exhibited for the PL mixture (40% decrease; p < 0.05, one-way ANOVA and Tukey’s multiple comparison, PL mixture versus POPC, DPPC, SDS and DOPC). With these magnitudes of fluorescence decline, return of fluorescence intensity was observed; during the ensuing 16 hour incubation period fluorescence reached levels that were 82 to 92% of the starting fluorescence intensities. Thus, when A2E was distributed amongst liposomes composed of DOPC, POPC, and DPPC in the lower phase, fluorescence in this layer was always decreased by 10–30% immediately after 430 nm irradiation. With SDS-A2E, the decrease in fluorescence was 30% and recovery of fluorescence reached 87% (±1.0%) of starting levels. In PBS, fluorescence declined after irradiation and continued to decrease, albeit at a slower pace, during the 16 hour incubation.

In contrast to the movement of A2E from oil to lipid and SDS phases, A2E fluorescence in a PBS/1%DMSO mixture was decreased after irradiation and further declined after 16 hour incubation with no indication of fluorescence recovery. This finding could indicate preferred movement of A2E from the lower DMSO/PBS phase to the upper oil phase.

We also tested A2E stability (50 μM) in DMSO-PBS (0.5%) during a 16 hour incubation at room temperature with no irradiation. By HPLC quantitation of peak area, A2E was found to be reduced by 9.7% (peak area, 0 time: 2.7 X 10^6^; after 16 hours 2.4 X 10^6^).

## Discussion

The fluorescence capability of A2E is enabled by an extensive system of carbon-carbon double bonds within its structure [16]. As with other fluorophores, the fluorescence efficiency of A2E is also subject to influences from surrounding microenvironmental effects. For instance, we previously observed that with excitation at 488 nm, the fluorescence intensity of A2E in methanol was less than half of that in chloroform [20]. Here too we found that in hydrophobic solvents such as toluene and chloroform, fluorescence intensity was greater and emission maxima were blue-shifted as compared to more polar solvents such as methanol and acetonitrile (Fig 1). It was also shown that different phospholipid environments confer varying levels of A2E fluorescence intensity [21]. Similarly, in the present work, initial levels of fluorescence (Fig 4) observed in the various phospholipid milieu differed, indicating that the fluorescence efficiency varied with the vesicle composition. When compared to the other milieu, fluorescence intensity of A2E in DOPC liposomes was greater than in SDS micelles and DMSO and accordingly was greater than in methanol and acetonitrile.

A2E is not only a fluorophore: it can also both generate and chemically quench singlet oxygen [16]. The loss of a double bond in an A2E molecule due to addition of singlet oxygen results in a change in fluorescence. For instance, A2E that has been photooxidized and carries one or two endoperoxide (peroxy-A2E) moieties (Fig 2E) exhibits an autofluorescence efficiency that is considerably increased in intensity relative to the parent A2E molecule [7, 16, 22]. If the oxidation occurs on the short arm of A2E, this increase in fluorescence can occur without a change in the absorbance/excitation maximum in the visible spectrum. Conversely, photooxidation on the long arm of the A2E molecule is associated with both hypsochromic shifts in the visible spectrum absorbance and with increased fluorescence efficiency. Further photooxidation of A2E is associated with progressive hypsochromic shifts in absorbance, a loss of ability to fluoresce, and eventually photo-induced chemical destruction. The destruction of A2E-associated photobleaching yields manifold fragmentation products bearing aldehydes and carbonyls [10, 23]. These relatively small photodegradation fragments do not absorb in the visible spectrum and thus are also colorless. With A2E photodegradation, the fluorescence emission spectra exhibit reduced intensity but the shape of the emission spectrum does not change appreciably (Fig 1) thus it is unlikely that new photoproducts having similar fluorescence emission are generated from reactions of the photofragments. These same photochemical observations have been made for all-*trans*-retinal dimer [16].

Since A2E photobleaching occurs subsequent to singlet oxygen addition at A2E carbon-carbon double bonds, and singlet oxygen formation requires the transfer of energy to ground state oxygen, photobleaching rates can vary with levels of ground state oxygen [24]. Thus, the availability of the latter could explain some of our observations. For instance, the solubility of oxygen in water is approximately 2.0 X 10^−3^ M and in DMSO is 0.33 X 10^−3^ M. Accordingly, we observed A2E to be most susceptible to photodegradation in PBS with 1% DMSO while the rate of photodegradation in 100% DMSO was less pronounced. Since oxygen molecules are nonpolar, the solubility of oxygen is generally greater in apolar organic solvents than in water. Thus in the solvents having higher relative permittivity and thus being relatively more oxygen limited, the rates of photodegradation were lower (Fig 2). Singlet oxygen lifetimes are also important to consider; for instance, considerable difference exists in the lifetime of singlet oxygen in methanol (7 μs) versus chloroform (247 μs) due to physical quenching of singlet oxygen in the former [25]. In the lipid interior of cell membranes, singlet oxygen has a lifetime of about twice as long as in an aqueous environment.

The amphiphilic structure of A2E accounts for its ability to aggregate [14, 26]. Aggregation of A2E in an aqueous/1% DMSO mixture, as utilized in this work, favored A2E photodegradation probably by bringing together the hydrophobic moieties of A2E and thus increasing the availability of polyene structures for singlet oxygen attack. The photo-induced loss of A2E was also greater in a mixture of PBS and 1% DMSO than in 100% DMSO. Conversely when A2E, which carries a positively charged head-group, was allowed to assemble into micelles with the anionic detergent SDS, A2E photodegradative loss was inversely correlated with SDS concentration. Perhaps this occurred because of the distance between molecules imposed by the intercalation of SDS. Since singlet oxygen is likely generated close to the moieties with which it reacts, the distance introduced between molecules of A2E may have limited the reactivity of singlet oxygen with the polyene chromophores of A2E.

In the simplified artificial bilayers we employed, differences in the rate of A2E photooxidative loss were also observed. All of the vesicles consisting of a single phospholipid species had the same head group (PC). Thus the differences we observed likely reflected varying acyl chain interactions. The double bond-containing arms of A2E, its wedge-shaped structure [14] and its bulky polar head group could cause A2E to behave differently in a tightly packed gel-phase provided by POPC as compared to the fluid phase of DOPC [27]. Photooxidative loss of A2E was more pronounced in the gel-phase wherein there was likely greater A2E aggregation. An additional factor influencing A2E incorporation into the vesicles composed of the PL mixture (PC, PS and PE) could have been the attraction between the cationic head group of A2E and the negatively charged head group of phosphatidylserine [26].

At the molecular level, oxidation of a double bond is an irrecoverable structural change [28]; thus a chemical reversal that reforms the original polyene structure of A2E cannot account for recovery of autofluorescence. Accordingly, we found that the fluorescence signal did not recover if the bleached fluorophore population was not replenished by diffusion from the adjacent oil fraction. On the other hand, with relocation of A2E from the A2E/oil mixture to the A2E/phospholipid vesicle fraction, we found that the partial return of autofluorescence was associated with increased measurable levels of A2E. Thus, as with photosensitizers that are used in photodynamic therapies and that undergo photoinduced displacement in cells resulting in changes in fluorescence characteristics [29], we suggest that movement of fluorophore to compensate for loss through photodegradation may underlie the recovery of autofluorescence after photobleaching.

One limitation of this work was the necessity of limiting the study to only a single lipofuscin fluorophore, although *in vivo*, RPE lipofuscin is a mixture of bisretinoids all of which emit fluorescence with a maximum wavelength of approximately 600 nm. While only A2E was employed here, the other identified bisretinoids of RPE lipofuscin exhibit similar photoreactive properties [7, 30, 31]; thus these findings likely apply to the bisretinoids of RPE in general. Other known bisretinoids of lipofuscin have longer wavelength absorbances (e.g. all-*trans*-retinal dimer-phosphatidyl-ethanolamine (λ_max_ 290, 510 nm) and thus could account for the photobleaching observed in response to 568 nm [1, 2].

**Fig 6:**
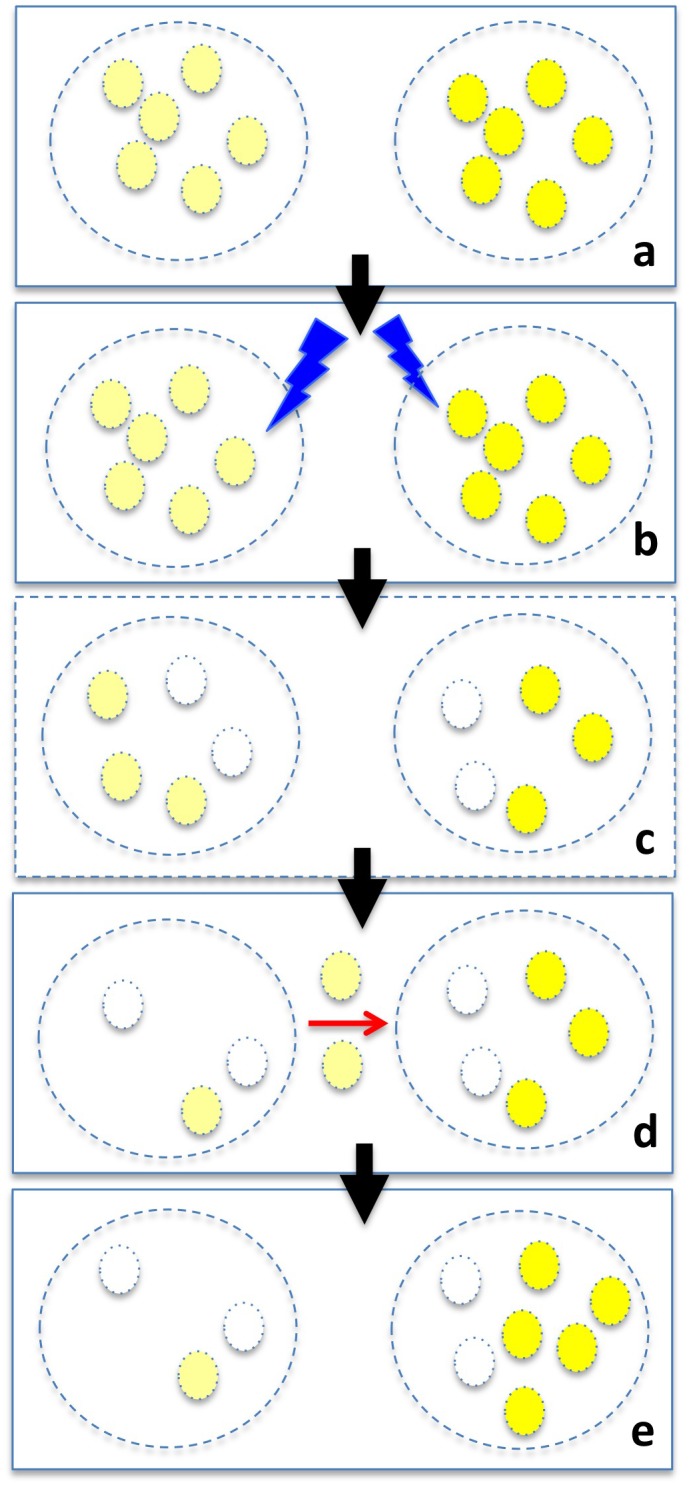
Model of a mechanism proposed to explain RPE autofluorescence recovery. Fractions of RPE bisretinoid (small circles) may be located in microenvironments (large dashed circles) that differ in their tendency to favor fluorescence emission (bright and light yellow) (a). Light exposure (b) can lead to loss of autofluorescence (bleaching) due to photooxidation (blank small circles) and photodegradation (elimination of circles) (c). Subsequently, molecules of bisretinoid within less fluorescent fractions may move (d) to microenvironments enabling greater fluorescence emission (e).

The lysosomal milieu in which A2E accumulates can be expected to be complex with a variety of solutes and polar and hydrophobic side-chains of proteins in a phospholipid-encased organelle. Thus any or all of the microenvironments discussed here would likely apply to the environment of bisretinoid compounds sequestered in lysosomes. Taken together our findings indicate that both the fluorescence intensity of A2E and its tendency toward photooxidation/photodegradation are more pronounced in nonpolar environments. This finding may also indicate that fluorescent and less-fluorescent fractions of intracellular A2E, do not generate singlet oxygen and photodegrade at the same rate. Accordingly, a model is proposed (Fig 6) in which a less fluorescent fraction of A2E that was also more protected from photodegradation could be held in reserve and thus available to replenish A2E associated fluorescence lost due to photodestruction. The recovery of fluorescence intensities after photobleaching is likely attributable to movement of A2E between microdomains of the lysosomal compartment of RPE. Bisretinoid photobleaching and RPE photodamage both occur due to photooxidation and photodegradation. We suggest that the magnitudes of these processes vary along a continuum. At the lower end of the continuum, photooxidation and photodegradation is evinced as fluorescence photobleaching in the absence of a threat to cell viability. At these light exposures, return of fluorescence can occur. At the upper end of the continuum, photooxidation/photodegradation of the fluorophore also presents as photobleaching, but the photo-destruction of the fluorophore can be sufficient to cause cellular damage. The process of photodegradation is damaging to RPE cells because of the release of aldehyde-bearing molecular fragments [10]. Amongst these fragments are the small dicarbonyls methylglyoxal and glyoxal that are known to react with and modify the activities of proteins [23]. These modified proteins can also incite inflammatory processes and, given their presence in drusen [32, 33], probably represent a connection between RPE bisretinoid lipofuscin and the formation of sub-RPE deposits. As shown in *in vitro* models, A2E and all-*trans*-retinal dimer also incite complement activation [34]. Since photooxidized forms of A2E and all-t*rans*-retinal dimer are detected in isolated human and mouse RPE, photooxidative processes are clearly ongoing in the RPE [7, 16]. The results reported here together with previous findings [1, 4] likely explain RPE autofluorescence bleaching and recovery. These studies elucidate mechanisms underlying autofluorescence photobleaching and photodamage, further our understanding of RPE lipofuscin photoreactivity, and could lead to the development of novel therapeutics aimed at making lipofuscin occupancy within the lysosome benign.
